# Supplementary material for: A chemokine-fusion vaccine targeting immature dendritic cells elicits elevated antibody responses to malaria sporozoites in infant macaques
Source: Sci Rep. 2021 Jan 13;11:1220. doi: 10.1038/s41598-020-79427-3 (PMC7807052; doi:10.1038/s41598-020-79427-3)
Supplement: Supplementary file 1 — Supplementary Figure S1. [file 41598_2020_79427_MOESM1_ESM.pdf]

Supplementary Materials

A Chemokine-Fusion Vaccine Targeting Immature Dendritic Cells Elicits Elevated Antibody Responses to Malaria Sporozoites in Infant Macaques

Kun Luo*, James T. Gordy*, Fidel Zavala* and Richard B. Markham*#

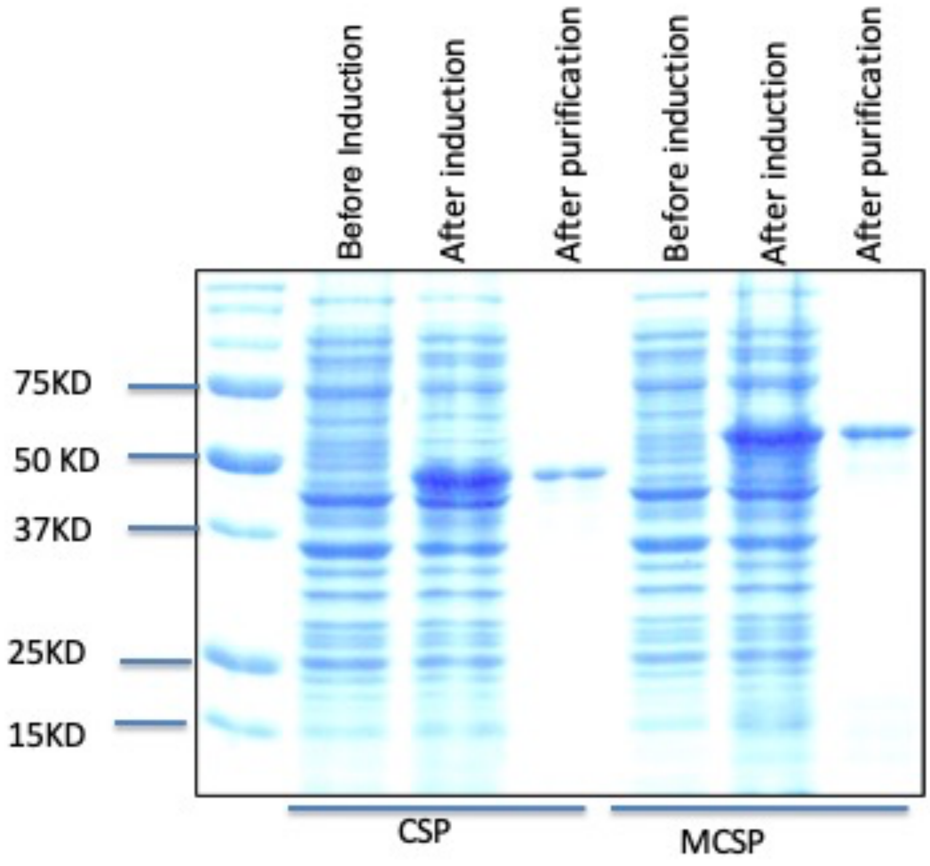

Figure S1. Polyacrylamide gel electrophoresis analysis of purified vaccine constructs. Protein samples were separated by 12% SDS- PAGE for visualization with Coomassie brilliant blue staining.
